# (*E*)-Ethyl 2-cyano-3-(1*H*-pyrrol-2-yl)acrylate

**DOI:** 10.1107/S1600536811028790

**Published:** 2011-07-23

**Authors:** Haldorai Yuvaraj, D. Gayathri, Rajesh G. Kalkhambkar, Vivek K. Gupta, Rajnikant

**Affiliations:** a School of Chemical Engineering, Yeungnam University, Gyeongsan, Gyeongbuk 712-749, Republic of Korea; b Department of Physics, Dr. M.G.R Educational and Research Institute University, Periyar E.V.R High Road, Maduravoyal, Chennai 600 095, India; c Department of Chemistry, Karnatak University’s Karnatak Science College, Dharwad 580 001, Karnataka, India; d Post Graduate Department of Physics & Electronics, University of Jammu, Jammu Tawi 180 006, India

## Abstract

All the non-H atoms of the title compound, C_10_H_10_N_2_O_2_, are nearly in the same plane with a maximum deviation of 0.093 (1) Å. In the crystal, adjacent mol­ecules are linked by pairs of inter­molecular N—H⋯O hydrogen bonds, generating inversion dimers with *R*_2_^2^(14) ring motifs.

## Related literature

For background to and applications of pyrrole derivatives, see: Fischer & Orth (1934 ▶). For the Knoevenagel condensation reaction and its applications, see: Knoevenagel (1898 ▶); Bigi *et al.* (1999 ▶). For the synthesis of related compounds, see: Knizhnikov *et al.* (2007 ▶); Sarda *et al.* (2009 ▶). For related structures, see: Ye *et al.* (2009 ▶); Wang & Jian (2008 ▶); Zhang *et al.* (2009 ▶).
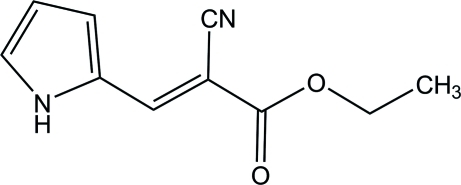


## Experimental

### 

#### Crystal data

C_10_H_10_N_2_O_2_
*M* *_r_* = 190.20
Monoclinic, 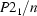

*a* = 6.2811 (2) Å
*b* = 9.4698 (3) Å
*c* = 16.3936 (5) Å
β = 92.645 (3)°
*V* = 974.06 (5) Å^3^
*Z* = 4
Mo *K*α radiation
μ = 0.09 mm^−1^
*T* = 293 K
0.30 × 0.20 × 0.15 mm

#### Data collection

Oxford Diffraction Xcalibur Sapphire3 diffractometer
Absorption correction: multi-scan (*CrysAlis PRO*; Oxford Diffraction, 2010 ▶) *T*_min_ = 0.971, *T*_max_ = 0.986
18157 measured reflections
1908 independent reflections
1574 reflections with *I* > 2σ(*I*)
*R*_int_ = 0.032

#### Refinement

*R*[*F* ^2^ > 2σ(*F* ^2^)] = 0.039
*wR*(*F* ^2^) = 0.113
*S* = 1.06
1908 reflections
128 parameters
H-atom parameters constrained
Δρ_max_ = 0.12 e Å^−3^
Δρ_min_ = −0.19 e Å^−3^

### 

Data collection: *CrysAlis PRO* (Oxford Diffraction, 2010 ▶); cell refinement: *CrysAlis PRO*; data reduction: *CrysAlis PRO*; program(s) used to solve structure: *SHELXS97* (Sheldrick, 2008 ▶); program(s) used to refine structure: *SHELXL97* (Sheldrick, 2008 ▶); molecular graphics: *PLATON* (Spek, 2009 ▶); software used to prepare material for publication: *SHELXL97*.

## Supplementary Material

Crystal structure: contains datablock(s) I, global. DOI: 10.1107/S1600536811028790/is2752sup1.cif

Structure factors: contains datablock(s) I. DOI: 10.1107/S1600536811028790/is2752Isup2.hkl

Supplementary material file. DOI: 10.1107/S1600536811028790/is2752Isup3.cml

Additional supplementary materials:  crystallographic information; 3D view; checkCIF report

## Figures and Tables

**Table 1:** Hydrogen-bond geometry (Å, °)

*D*—H⋯*A*	*D*—H	H⋯*A*	*D*⋯*A*	*D*—H⋯*A*
N1—H1*A*⋯O1^i^	0.86	2.09	2.874 (2)	151

Symmetry code: (i) 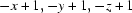.

**Fig. 1.**
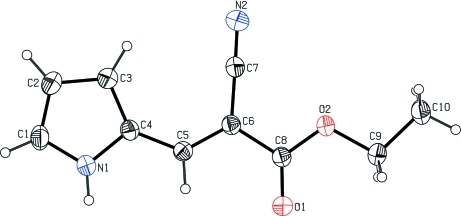
The molecular structure of the title compound, showing 30% probability displacement ellipsoids.

**Fig. 2.**
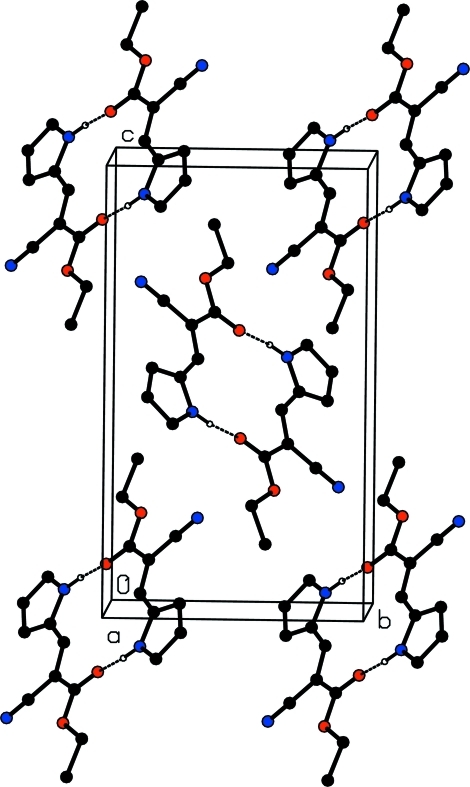
A molecular packing view of the title compound, showing intermolecular interactions. For clarity, hydrogen atoms which are not involved in hydrogen bonding have been omitted.

## supplementary crystallographic information

### Comment

The chemistry of pyrrole compounds and biological activities of the related compounds has been extensively studied (Fischer & Orth, 1934). The Knoevenagel condensation is an important carbon–carbon bond forming reaction in organic synthesis (Knoevenagel, 1898). Ever since its discovery, the Knoevenagel reaction has been widely used in organic synthesis to prepare coumarins and their derivatives, which are important intermediates in the synthesis of cosmetics, perfumes and pharmaceuticals (Bigi *et al.*, 1999). With the view of biological importance the title compound was synthesized and reported here its crystal structure.

Bond lengths and bond angles are comparable with the similar crystal structures solved earlier (Ye *et al.*, 2009; Wang & Jian, 2008; Zhang *et al.*, 2009). All the non-hydrogen atoms in the molecule are nearly in the same plane with the maximum out-of-plane deviation of 0.093 (1) Å (r.m.s. deviation = 0.04 Å). The crystal packing is stabilized by N—H···O intermolecular interactions, generating a centrosymmetric dimer of *R*_2_^2^(14) ring.

### Experimental

A solution of pyrrole-2-aldehyde (1 mol), ethyl cyanoacetate (1.2 mol) and piperidine (0.1 ml) in ethanol (20 ml) was stirred at room temperature for 8 h. After removal of the volatiles *in vacuo*, orange solid was obtained in quantitative yield. A sample for analysis was obtained by recrystallization from EtOAc as pale yellow needles: ^1^H NMR (300 MHz, CDCl_3_) δ p.p.m.: 1.38 t (3*H*, CH_3_), 4.35 q (2*H*, CH_2_), 6.41 m (1*H*, CH), 6.92 m (1*H*, CH), 7.22 m (1*H*, CH), 7.98 s (1*H*, HC=C), 9.92 s (1*H*, NH).

### Refinement

All H atoms were refined using a riding model, with *d*(C—H) = 0.93 Å for aromatic, 0.97 Å for CH_2_ and  0.96 Å for CH_3_, and *d*(N—H) = 0.86 Å, and with *U*_iso_(H) = 1.2*U*_eq_(C, N) or 1.5*U*_eq_(methylC)

### Crystal data

**Table:**

C_10_H_10_N_2_O_2_	*F*(000) = 400
*M**_r_* = 190.20	*D*_x_ = 1.297 Mg m^−^^3^
Monoclinic, *P*2_1_/*n*	Mo *K*α radiation, λ = 0.71073 Å
Hall symbol: -P 2yn	Cell parameters from 7544 reflections
*a* = 6.2811 (2) Å	θ = 3.5–29.0°
*b* = 9.4698 (3) Å	µ = 0.09 mm^−^^1^
*c* = 16.3936 (5) Å	*T* = 293 K
β = 92.645 (3)°	Rectangular, light yellow
*V* = 974.06 (5) Å^3^	0.30 × 0.20 × 0.15 mm
*Z* = 4	

### Data collection

**Table:**

Oxford Diffraction Xcalibur Sapphire3 diffractometer	1908 independent reflections
Radiation source: fine-focus sealed tube	1574 reflections with *I* > 2σ(*I*)
graphite	*R*_int_ = 0.032
Detector resolution: 16.1049 pixels mm^-1^	θ_max_ = 26.0°, θ_min_ = 3.5°
ω scans	*h* = −7→7
Absorption correction: multi-scan (*CrysAlis PRO*; Oxford Diffraction, 2010)	*k* = −11→11
*T*_min_ = 0.971, *T*_max_ = 0.986	*l* = −20→20
18157 measured reflections	

### Refinement

**Table:**

Refinement on *F*^2^	Primary atom site location: structure-invariant direct methods
Least-squares matrix: full	Secondary atom site location: difference Fourier map
*R*[*F*^2^ > 2σ(*F*^2^)] = 0.039	Hydrogen site location: inferred from neighbouring sites
*wR*(*F*^2^) = 0.113	H-atom parameters constrained
*S* = 1.06	*w* = 1/[σ^2^(*F*_o_^2^) + (0.0623*P*)^2^ + 0.1138*P*] where *P* = (*F*_o_^2^ + 2*F*_c_^2^)/3
1908 reflections	(Δ/σ)_max_ < 0.001
128 parameters	Δρ_max_ = 0.12 e Å^−^^3^
0 restraints	Δρ_min_ = −0.19 e Å^−^^3^

### Special details

**Table:**

Geometry. All e.s.d.'s (except the e.s.d. in the dihedral angle between two l.s. planes) are estimated using the full covariance matrix. The cell e.s.d.'s are taken into account individually in the estimation of e.s.d.'s in distances, angles and torsion angles; correlations between e.s.d.'s in cell parameters are only used when they are defined by crystal symmetry. An approximate (isotropic) treatment of cell e.s.d.'s is used for estimating e.s.d.'s involving l.s. planes.
Refinement. Refinement of *F*^2^ against ALL reflections. The weighted *R*-factor *wR* and goodness of fit *S* are based on *F*^2^, conventional *R*-factors *R* are based on *F*, with *F* set to zero for negative *F*^2^. The threshold expression of *F*^2^ > σ(*F*^2^) is used only for calculating *R*-factors(gt) *etc*. and is not relevant to the choice of reflections for refinement. *R*-factors based on *F*^2^ are statistically about twice as large as those based on *F*, and *R*- factors based on ALL data will be even larger.

### Fractional atomic coordinates and isotropic or equivalent isotropic displacement parameters (Å^2^)

**Table:**

	*x*	*y*	*z*	*U*_iso_*/*U*_eq_	
C1	−0.1517 (2)	0.73171 (17)	0.56284 (9)	0.0557 (4)	
H1	−0.2260	0.7170	0.6099	0.067*	
C2	−0.2045 (3)	0.82705 (18)	0.50230 (9)	0.0596 (4)	
H2	−0.3208	0.8879	0.5007	0.072*	
C3	−0.0526 (2)	0.81600 (16)	0.44393 (9)	0.0522 (4)	
H3	−0.0488	0.8687	0.3962	0.063*	
C4	0.0926 (2)	0.71274 (13)	0.46903 (7)	0.0403 (3)	
C5	0.2764 (2)	0.65298 (13)	0.43588 (8)	0.0403 (3)	
H5	0.3441	0.5838	0.4679	0.048*	
C6	0.36856 (19)	0.68137 (13)	0.36460 (7)	0.0390 (3)	
C7	0.2877 (2)	0.78645 (15)	0.30869 (8)	0.0433 (3)	
C8	0.5592 (2)	0.60070 (14)	0.34340 (7)	0.0400 (3)	
C9	0.8147 (2)	0.56123 (16)	0.24473 (8)	0.0502 (4)	
H9A	0.7809	0.4614	0.2415	0.060*	
H9B	0.9363	0.5737	0.2827	0.060*	
C10	0.8645 (3)	0.61655 (18)	0.16209 (9)	0.0593 (4)	
H10A	0.7448	0.6008	0.1247	0.089*	
H10B	0.9870	0.5683	0.1429	0.089*	
H10C	0.8937	0.7159	0.1657	0.089*	
N1	0.02503 (19)	0.66332 (12)	0.54287 (7)	0.0472 (3)	
H1A	0.0873	0.5982	0.5717	0.057*	
N2	0.2208 (2)	0.87126 (15)	0.26485 (8)	0.0631 (4)	
O1	0.63859 (15)	0.50906 (11)	0.38609 (6)	0.0539 (3)	
O2	0.63298 (14)	0.64028 (10)	0.27206 (5)	0.0454 (3)	

### Atomic displacement parameters (Å^2^)

**Table:**

	*U*^11^	*U*^22^	*U*^33^	*U*^12^	*U*^13^	*U*^23^
C1	0.0510 (8)	0.0684 (10)	0.0490 (8)	0.0055 (7)	0.0159 (6)	−0.0045 (7)
C2	0.0537 (9)	0.0672 (10)	0.0587 (9)	0.0183 (7)	0.0105 (7)	0.0000 (8)
C3	0.0544 (8)	0.0562 (9)	0.0464 (8)	0.0119 (7)	0.0072 (6)	0.0048 (6)
C4	0.0420 (7)	0.0431 (7)	0.0359 (6)	−0.0008 (6)	0.0034 (5)	−0.0025 (5)
C5	0.0402 (7)	0.0420 (7)	0.0386 (7)	0.0013 (5)	0.0018 (5)	−0.0001 (5)
C6	0.0375 (7)	0.0422 (7)	0.0375 (6)	−0.0014 (5)	0.0024 (5)	0.0005 (5)
C7	0.0414 (7)	0.0477 (8)	0.0412 (7)	0.0014 (6)	0.0068 (5)	0.0012 (6)
C8	0.0388 (7)	0.0434 (7)	0.0380 (7)	−0.0012 (5)	0.0032 (5)	0.0002 (5)
C9	0.0450 (7)	0.0541 (8)	0.0526 (8)	0.0072 (6)	0.0133 (6)	0.0026 (7)
C10	0.0608 (9)	0.0625 (10)	0.0565 (9)	0.0038 (7)	0.0227 (7)	0.0043 (7)
N1	0.0487 (7)	0.0524 (7)	0.0412 (6)	0.0062 (5)	0.0091 (5)	0.0043 (5)
N2	0.0660 (9)	0.0669 (8)	0.0570 (8)	0.0129 (7)	0.0092 (6)	0.0184 (7)
O1	0.0540 (6)	0.0611 (6)	0.0474 (6)	0.0163 (5)	0.0092 (4)	0.0123 (5)
O2	0.0425 (5)	0.0507 (6)	0.0437 (5)	0.0047 (4)	0.0120 (4)	0.0066 (4)

### Geometric parameters (Å, °)

**Table:**

C1—N1	1.3386 (18)	C6—C8	1.4753 (18)
C1—C2	1.371 (2)	C7—N2	1.1447 (17)
C1—H1	0.9300	C8—O1	1.2080 (15)
C2—C3	1.386 (2)	C8—O2	1.3316 (15)
C2—H2	0.9300	C9—O2	1.4528 (16)
C3—C4	1.3869 (19)	C9—C10	1.4991 (19)
C3—H3	0.9300	C9—H9A	0.9700
C4—N1	1.3827 (16)	C9—H9B	0.9700
C4—C5	1.4165 (18)	C10—H10A	0.9600
C5—C6	1.3546 (18)	C10—H10B	0.9600
C5—H5	0.9300	C10—H10C	0.9600
C6—C7	1.4301 (18)	N1—H1A	0.8600
			
N1—C1—C2	108.49 (12)	O1—C8—O2	124.05 (12)
N1—C1—H1	125.8	O1—C8—C6	123.60 (11)
C2—C1—H1	125.8	O2—C8—C6	112.35 (11)
C1—C2—C3	107.40 (13)	O2—C9—C10	107.37 (12)
C1—C2—H2	126.3	O2—C9—H9A	110.2
C3—C2—H2	126.3	C10—C9—H9A	110.2
C2—C3—C4	108.22 (13)	O2—C9—H9B	110.2
C2—C3—H3	125.9	C10—C9—H9B	110.2
C4—C3—H3	125.9	H9A—C9—H9B	108.5
N1—C4—C3	105.90 (12)	C9—C10—H10A	109.5
N1—C4—C5	119.30 (12)	C9—C10—H10B	109.5
C3—C4—C5	134.80 (12)	H10A—C10—H10B	109.5
C6—C5—C4	129.78 (12)	C9—C10—H10C	109.5
C6—C5—H5	115.1	H10A—C10—H10C	109.5
C4—C5—H5	115.1	H10B—C10—H10C	109.5
C5—C6—C7	122.53 (12)	C1—N1—C4	110.00 (12)
C5—C6—C8	118.95 (11)	C1—N1—H1A	125.0
C7—C6—C8	118.53 (11)	C4—N1—H1A	125.0
N2—C7—C6	178.93 (14)	C8—O2—C9	115.85 (10)
			
N1—C1—C2—C3	−0.38 (18)	C7—C6—C8—O1	−179.34 (12)
C1—C2—C3—C4	0.31 (18)	C5—C6—C8—O2	−179.66 (11)
C2—C3—C4—N1	−0.12 (16)	C7—C6—C8—O2	0.57 (17)
C2—C3—C4—C5	178.72 (15)	C2—C1—N1—C4	0.31 (18)
N1—C4—C5—C6	178.23 (12)	C3—C4—N1—C1	−0.11 (16)
C3—C4—C5—C6	−0.5 (3)	C5—C4—N1—C1	−179.17 (12)
C4—C5—C6—C7	1.1 (2)	O1—C8—O2—C9	2.78 (19)
C4—C5—C6—C8	−178.64 (12)	C6—C8—O2—C9	−177.13 (10)
C5—C6—C8—O1	0.4 (2)	C10—C9—O2—C8	176.96 (11)

### Hydrogen-bond geometry (Å, °)

**Table:**

*D*—H···*A*	*D*—H	H···*A*	*D*···*A*	*D*—H···*A*
N1—H1A···O1^i^	0.86	2.09	2.874 (2)	151

Symmetry codes: (i) −*x*+1, −*y*+1, −*z*+1.
